# The Week's Parliament and Publications

**Published:** 1910-08-06

**Authors:** 


### The Week's Parliament and Publications.

### ROYAL COLLEGE OF SURGEONS, IRELAND.

In reply to Mr. Long, the Chancellor of the Exchequer said he would be prepared to give careful consideration to any representations on behalf of the Royal College of Surgeons, Ireland, which the Chief Secretary might think fit to make to him on the subject. When the Irish Universities Act, 1908, was under discussion in the House of Commons a claim was pressed in favour of an annual grant of £5,000 to the College.

### A RARE COMPLICATION OF VACCINATION.

In reply to Sir William Collins, who asked whether any inquiry was instituted in regard to the death of Mary Ellen Thody, of Penge, which occurred on March 18, and which was certified to be due to vaccination and septic poisoning, the President of the Local Government Board replied that an inquiry was instituted. The child was vaccinated by a private practitioner; the condition following vaccination appears to have been generalised vaccinia, an extremely rare complication of vaccination.

### MIDWIVES BILL.

The Midwives (No. 2) Bill was read a third time in the House of Lords on Monday, August 1. Several amendments were made in committee and on report, and as the Bill now stands provision is made for the presence on the Central Midwives Board of a person appointed by the Royal British Nurses' Association; only one of the two persons appointed by the Incorporated Midwives Institute need be a certified midwife. Clause 12 has been amended so as to make eligible for certification any woman who produces evidence that she is qualified to be appointed as a midwife by a Board of Guardians in Ireland.

### OUTPUT OF SCIENTIFIC INSTRUMENTS AND APPLIANCES IN THE UNITED KINGDOM.

Further preliminary tables summarising the results of the Returns received under the Census of Production Act, 1906, have been published. These deal with a number of industries, among which is the manufacture of scientific instruments, apparatus, appliances, and accessories. The tables relate to a period of twelve months. The average number of persons (other than outworkers) employed in factories and workshops engaged in the manufacture of scientific instruments, apparatus, and appliances is returned as 14,122, of whom 10,337 were males and 3,785 females. The gross output or selling value of work done amounted to £2,526,000, the cost of materials used £993,000. The net output amounted to £1,507,000. The value of the output of surgical and dental instruments, apparatus, and appliances was £329,000; artificial teeth and dentists' materials, £274,000; surgical and medical bandages and dressings, £57,000; optical instruments and appliances (including spectacles, ophthalmoscopes, etc.), £241,000.

### NEWCASTLE-ON-TYNE WORKHOUSE HOSPITAL.

Mr. Hudson asked the President of the Local Government Board if he was aware that a number of the patients in the workhouse hospital, Newcastle-on-Tyne, had been compelled to lie on the floor of the wards owing to lack of accommodation; and, if so, whether he was prepared to take any steps to remedy the matter. Mr. Burns replied that he was aware that the accommodation in the workhouse infirmary was insufficient, and he had for some time been in correspondence with the Guardians on the subject. A further letter pressing the Guardians to deal with the question immediately was sent to them last Friday.

### INFANT AND CHILD MORTALITY.

A report on infant and child mortality has been published as a supplement to the report of the Medical Officer of the Local Government Board. The object of the report is to determine whether reduction of infant mortality implies any untoward influence on the health of survivors to later years; to indicate the communities which are characterised by a continuing high rate of infant mortality; and to assess, so far as is possible, the relative value of the different factors of excessive infant mortality. In Part I. the relationship of infant mortality to mortality at higher ages is discussed. In Part II. centres where there is a continuance of high mortality are enumerated. In Part III. an attempt is made to set out the mutual responsibility of local authorities and of parents in the continuance of excessive infant mortality.—Report on "Infant and Child Mortality" (Cd. 5263), price 1*s.* 3*d.*

### ST MARY'S HOSPITAL BACTERIOLOGICAL DEPARTMENT.

Mr. Lynch asked the Chancellor of the Exchequer whether any portion of the grant to Universities might be made available to the direct encouragement of scientific research; and whether, in particular, in view of the high character of original work carried out at St. Mary's Hospital bacteriological department, and in view also of the acknowledged importance of these results in combating tuberculosis he would take steps to ensure the removal of the financial embarrassment which threatens to impede the work of that institution. In reply, Mr. Lloyd George stated that the grant made annually by Parliament in aid of University Colleges was distributed on the recommendation of the advisory committee, and the committee had always paid special attention to the post-graduate and research work of colleges applying for a share of the grant. Any institution which desired to participate in the grant, and is prepared to satisfy the conditions laid down in the Treasury Minute of June 3, 1909 (No. 182 of 1909), should make application in the first instance to the Advisory Committee.

